# Ursolic acid silences CYP19A1/aromatase to suppress gastric cancer growth

**DOI:** 10.1002/cam4.4536

**Published:** 2022-05-11

**Authors:** Wen‐Lung Ma, Ning Chang, Yingchun Yu, Yu‐Ting Su, Guan‐Yu Chen, Wei‐Chung Cheng, Yang‐Chang Wu, Ching‐Chia Li, Wei‐Chun Chang, Juan‐Cheng Yang

**Affiliations:** ^1^ Graduate Institute of Biomedical Sciences Center for Tumor Biology Department of Pharmacology Chinese Medicine Research Center Drug Development Center, and Graduate Institute of Chinese Medicine Graduate Institute of Integrated Medicine School of Medicine China Medical University Taichung Taiwan; ^2^ Department of Medical Research Chinese Medicine Research and Development Center, and Department of Obstetrics & Gynecology China Medical University Hospital Taichung Taiwan; ^3^ Department of Nursing Department of Biotechnology College of Medical and Health Science Asia University Taichung Taiwan; ^4^ Graduate Institute of Clinical Medicine College of Medicine Kaohsiung Medical University Kaohsiung Taiwan; ^5^ Department of Urology Kaohsiung Medical University Hospital Kaohsiung Taiwan; ^6^ Department of Urology School of Medicine College of Medicine Kaohsiung Medical University Kaohsiung Taiwan; ^7^ Department of Obstetrics and Gynecology Asia University Hospital Taichung Taiwan; ^8^ School of Chinese Medicine China Medical University Taichung Taiwan

**Keywords:** Ar silencer, CYP19A1/aromatase, gastric cancer, *Hedyotis diffusa Willd*, ursolic acid

## Abstract

**Introduction:**

Gastric cancer (GCa) is a malignancy with few effective treatments. Ursolic acid (UA), a bioactive triterpenoid enriched in *Hedyotis diffusa* Willd, known to suppress GCa without identified target. CYP19A1 (cytochrome P450 family 19A1; also known as aromatase, Ar) was correlated to GCa prognosis. Relatedly, Ar silencers, which halt the expression of Ar exhibited anti‐GCa effects in experimental models, are currently being investigated.

**Method:**

The docking simulation score of UA was compared with Ar inhibitors, e.g., letrozole, exemestane, in Ar protein crystallization. *Hedyotis diffusa* Willd ethanol extract, UA, or 5‐fluracil were applied onto AGS, SC‐M1, MKN45 GCa cells for cancer inhibition tests. Immunoblot for measuring gene expressions upon drug treatments, or gene knockdown/overexpression. Treatments were also applied in a MKN45 implantation tumor model. A web‐based GCa cohort for Ar expression association with prognosis was performed.

**Result:**

The ethanol extracts of *Hedyotis diffusa* Willd, enrich with UA, exhibited cytotoxic activity against GCa cells. Molecular docking simulations with the 3D Ar structure revealed an excellent fitting score for UA. UA increase cytotoxic, and suppressed colony, in addition to its Ar silencing capacity. Moreover, UA synergistically facilitated 5‐FU, (a standard GCa treatment) regimen in vitro. Consistent with those results, adding estradiol did not reverse the cancer‐suppressing effects of UA, which confirmed UA acts as an Ar silencer. Furthermore, UA exhibited tumor‐suppressing index (TSI) score of 90% over a 6‐week treatment term when used for single dosing in xenograft tumor model. In the clinical setting, Ar expression was found to be higher in GCa tumors than normal parental tissue from the TCGA (The Cancer Genome Atlas) cohort, while high Ar expression associated with poor prognosis. Together, the results indicate UA could be used to treat GCa by silencing Ar expression in GCa. *Hedyotis diffusa* Willd ethanol extract could be an functional food supplements.

## Graphical abstract

Ursolic acid (UA) has been discovered to inhibit gastric cancer without identified target. This article defined UA as is aromatase silencer, of which turn off aromatase expression rather than suppressing enzymatic activity.

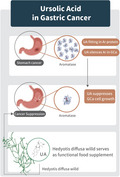

## 1 INTRODUCTION

Gastric cancer (GCa) is the fifth most commonly occurring cancer and ranks second in terms of cancer mobility among all cancers worldwide.^1^, ^2^, ^3^ The incidence of GCa varies worldwide,^4^, ^5^ but the disease has a poor prognosis in general, with a 5‐year survival rate of only 10% or less.^6^ Most GCa patients are diagnosed at an advanced stage and quickly relapse, that is, within 12 months, following surgery.^6^, ^7^, ^8^ Resection is the first‐choice treatment modality, yet high recurrence rates still occur following resection.^9^ Meanwhile, although chemotherapy is often effective against early stage GCa, patients with advanced GCa still have a poor prognosis when treated with chemotherapy.^4^, ^6^, ^9^ Therefore, there is currently a high clinical demand for new adjuvant or neoadjuvant chemotherapies for GCa.^10^ Among the various available chemoagents, 5‐fluorouracil (5‐FU)‐based chemotherapy drugs are considered as standard therapy.^11^ Therefore, with respect to new drug development, the therapeutic outcomes of 5‐FU treatment often serve as the baseline for comparison in GCa patients.^9^ Aside from 5‐FU treatments, studies have shown that 15% of GCa patients are HER2‐positive,^12^, ^13^ and these patients can be treated with a HER2 inhibitor as an alternative treatment once 5‐FU treatment has failed. However, most of the patients for whom 5‐FU has failed are HER2‐negative patients (only 5% are HER2‐positive),^14^ meaning there is a huge unmet medical need of additional treatments for such patients. One of the important obstacles for slowing progression of the development for GCa therapeutics is the lacking of theranostic marker.^4^, ^15^

One previous study has shown that ~20% of GCa patients overly express CYP19A1 (cytochrome P450 family 19 subfamily A member 1; also known as aromatase, Ar).^16^ Because Ar acts, in terms of biochemical function, as a metabolic enzyme to convert testosterone into estradiol,^17^ aromatase inhibitors have been developed and implemented as treatments for estrogen receptor (ESR)‐positive breast cancer patients.^18^ Unfortunately, the nonsteroidal Ar inhibitors (e.g., anastrozole and letrozole) exert little cytotoxic efficacy, whereas one steroidal Ar inhibitor, exemestane, has shown excellent cancer‐suppressive effects in a preclinical GCa model.^19^, ^20^ This discrepancy in the effects of various Ar inhibitors is due to the novel mechanisms of action of these drugs. For example, Yang et al.^20^ revealed that exemestane suppresses GCa cell growth by silencing Ar expression in the GCa cells. Therefore, finding new Ar silencers is an important task in the field of oncology.

In order to target Ar, we would like to find out novel compound to evaluate its possibility to suppress GCa cells. Small molecule compounds from natural products are important resources for new drug discovery and development. In the current study, we accessed 2073 compounds from the TDEC (Taiwan Database of Extracts and Compounds; https://tdec.kmu.edu.tw), and performed molecular docking simulations of these compounds into the Ar structure. Interestingly, the well‐known anticancer compound ursolic acid (UA) was found to have a high fitting score when used in such a simulation. Its outstanding fitting score trajectory indicated that it has mechanism of action similar to that of exemestane in suppressing GCa cells. The current report characterized pharmacological mode of action for the well‐known anticancer natural compound UA that can suppress GCa cell growth.

## 2 MATERIALS AND METHODS

### 2.1 Molecular docking for simulating UA in CYP19A1

To evaluate the interaction between small molecular inhibitors and CYP19A1, the molecular docking simulations were performed using AutoDock 4.2 with the Lamarckian genetic algorithm.^21^ The structural information of human placental aromatase (also known as CYP19A1; PDB ID: 3S7S) was obtained from the RCSB Protein Data Bank (https://www.rcsb.org/).^22^ We selected the PDB structure that co‐crystalizes with the breast cancer drug exemestane. For the calculation of binding energy, the co‐crystalized ligands were removed. Furthermore, the polar hydrogens and Kallman united atom charges were added into CYP19A1 for calculating the docking mode by the AutoDock Tool 1.5.6 interfaces (ADT).^23^ To optimize the small molecular inhibitors (letrozole, ursolic acid, and exemestane), we used the MMFF94 force field of the ChemBio3D software (version 11.0; Cambridge Soft Corp.). In addition, hydrogens and Gasteiger charges were added into the inhibitors for the docking by the ADT as well. A grid box with dimensions of 40 × 40 × 40 Å grid points at a spacing of 0.375 Å calculated by the AutoGrid program was centered at the coordinates of *x* = 86.57, *y* = 54.12, and *z* = 45.85. All the parameters for the docking were set to default except that the maximum number of energy evaluation increase was set to 25,000,000 per run. The results were analyzed with cluster analysis, and the simulation models were shown using the BIOVIA Discovery Studio 4.5 Visualizer (Dassault Systèmes, BIOVIA Corp.).^4^

### 2.2 Cell culture and reagents

The human gastric cancer cell lines AGS, SCM1, and MKN45 were purchased from the Food Industry Research and Development Institute in Hsinchu, Taiwan. AGS, SCM1, and MKN45 cells were cultured in RPMI 1640 medium (RPMI) (GIBCO) with 10% fetal calf serum and 1% penicillin/streptomycin (GIBCO). The cell lines were maintained at 37°C in a humidified atmosphere of 5% CO_2_. E2 (β‐estradiol: E2758), 5‐FU (F6627), and ursolic acid (U6753) were purchased from Sigma‐Aldrich. Stock solution of 5‐FU (10 mM), ursolic acid (100 mM), and 17β‐estradiol (10 mM) was prepared in DMSO and 99.5% ethanol. Aliquoting these stock solutions and frozen them at −20°C. CYP19A1 plasmid (Cat. # HG15323‐UT) and its control (pCMV3 vector: Cat. # CV011) were purchased from Sino Biological. Plasmids were transformed to DH5α competent cells and amplified in LB medium containing ampicillin (100 μg/ml) overnight using a 37°C shaker with speed at 180 rpm. Then the bacteria were harvested and plasmids were purified using Invitrogen plasmid extraction and purification kit according to the procedure provided by the manufacture.

### 2.3 Transient transfection

2.5 × 10^5^ cells were seeded in 6‐cm dish, and the transfection was performed next day. The transfection reagent is Lipofectamine 2000 (Invitrogen). The operations, respectively, according to manufacturer's instructions (Invitrogen or Promega). 3 microgram plasmid mixed with 20 microliter of Lipofectamine 2000 in 0.5 ml of Opti‐MEM reduced serum medium (Thermo Fisher). The DNA–lipofectamine mixture was incubated at room temperature for 15 min, then the mixture was added to the cells in a 6‐cm dish containing 1 ml of RPMI medium without fetal bovine serum and 1% antibiotics. After 6 h of transfection, the transfection medium was replaced by growth medium. Transfected cells were subjected to colony formation assay, cytotoxic assay, and western blotting 48–72 h later.

### 2.4 Cytotoxic analysis and IC50 calculation

The WST‐1 assay was used for assessing cell growth.^24^ Briefly, 2.5 × 10^3^ cells/well were seeded in 96‐well plates with RPMI/10% fetal bovine serum (FBS) and were incubated for 24 h. The drugs were then added following previous work,^20^ and the cells were further incubated (single treatment: UA [20, 40, 60, 80, and 100 μM] and 5‐FU [10 μM]). After 48 h of incubation, 1:10/v:v of WST‐1 (Roche) solution was added for a further 60‐min incubation period, after which the cell viability was measured through colorimetric detection with an ELISA plate reader (BECKMAN COULTER PARADIGM TM Detection Platform) at an absorbance of 450 nm to generate an optical density proportional to the relative abundance of live cells in the given wells. The values of 50% inhibitory concentration (IC50)^25^ for each drug were determined using CalcuSyn software^26^ (Biosoft).

### 2.5 Colony formation assay and standard cell number count

The method used for colony formation assay and standard cell number count was modified from a previous study.^27^ In brief, four sets of 500 cells/well were seeded in a 6‐well plates with RPM1/10% FBS and incubated for 24 h. Then, the cells in each set were treated, respectively, with the investigated drugs (vehicle, 5‐FU: 10 μM, UA) for 7 days. Next, 1000 μl of 4% formaldehyde solution was added to the fixed cells in each well, after which the mixture was incubated at room temperature for 1 h. Then, the cells were covered with crystal violet stain. After 1 h, the crystal violet was washed from the 6‐well plates, and the cell colonies were photographed. The cell numbers were then counted using ImageJ software.

### 2.6 Combination index analysis

The theory of linear and nonlinear regression analysis by Chou‐Talalay method was used to evaluate the synergy (CI < 1), additive (CI = 1), and antagonism (CI > 1) of the combination drug treatment.^25^ The combination index (CI) is calculated using the following formula: CA,X and CB,X are the concentrations of drugs A and B that achieve x% drug effect when used in combination. ICX,A and ICX,B are the concentrations for single agents to achieve the same effect.^28^ Combination index values were calculated using the CompuSyn software (CompuSyn Inc.).

CI=CA,x/ICx,A+CB,x/ICx,B

### 2.7 Total RNA isolation and CDNA synthesis

RNA was extracted from AGS, SCM1, and MKN45 cells as in a previous study.^29^ In each 100‐mm dish, 1 × 10^6^ cells/well were seeded with RPMI in 10% FBS and were then incubated for 24 h. The cells were then treated with UA (5, 10, and 15 μM) and were incubated at 37°C for a further 48 h. Cells that had reached 80%–90% confluence in the 100‐mm dishes were then lysed with 1 ml of Trizol (Invitrogen). Next, phenol/chloroform was added, and the RNA‐rich layers were separated by centrifugation. With the addition of 2‐propanol, soluble RNA was then precipitated. Next, the RNA was rinsed with 75% ethanol and dried at room temperature before being dissolved in RNase‐free water. For first‐strand cDNA synthesis, 5 μg of total RNA was used to perform reverse transcription PCR with the PrimeScript^TM^ RT reagent kit (TAKARA Bio Inc.), while synthesized miRNA cDNA was produced using the Mir‐X^TM^ miRNA First‐Strand synthesis Kit (Clontech). The synthesized cDNA and miRNA were produced following the manufacturers’ instructions.

### 2.8 Quantitative real‐time PCR analysis

A real‐time detection system (Bio‐Rad Laboratories, Inc.) and the KAPA^TM^ SYBR FAST One‐Step qRT‐PCR Kit (KAPABIOSYSTEMS) were used according to the manufacturers’ instructions. Relative gene expression was determined by normalizing the expression level of the target gene to the expression level of a housekeeping gene (actin). Threshold value (Ct) dynamics were used (2^−ΔΔCt^) for the quantitation of gene expression. The qRT‐PCR primer sequences used were as follows^20^: CYP19A1 forward 5′‐ACC CTT CTG CGT CGT GTC A‐3′, reverse 5′‐TCT GTG GAA ATC CTG CGT CTT‐3′.

### 2.9 Western blot

Cells were lysed in RIPA buffer (50 mM Tris, pH 7.4, 150 mM, NaCl, 5 mM EDTA, pH 8.0, 30 mM NaF, 1 mM Na3VO4, 40 mM β‐glycerophosphate, 0.1 mM PMSF, protease inhibitors, 10% glycerol, and 1% Nonidet‐P40). Forty micrograms of protein per lane was prepared in 10% SDS gel for electrophoresis, and then the proteins were transferred onto PVDF membranes. These membranes were stained with anti‐actin (sc‐47778, Santa Cruz) and anti‐aromatase (anti‐CYP19A1: sc‐374176, Santa Cruz) at 4°C overnight. Proteins were visualized and quantified using ChemiDoc system (Bio‐Rad Laboratories).

### 2.10 Animal model

Six‐week‐old male BALB/cAnN. Cg‐*Foxnl^nu^*/CrlNarl mice (National Laboratory Animal Center) were raised under specific pathogen‐free conditions (Laboratory Animal Center of China Medical University Hospital). The xenograft tumor model used was modified from that presented in a previous study.^30^ 1 × 10^7^ of MKN45 cells were subcutaneous (s.c.) injected into two sites on the lower back of each mouse. When the tumor volume reached 200 mm^3^, the mice were randomly assigned to three groups (placebo, 5‐FU (5 mg/kg^31^), and ursolic acid (20 mg/kg^32^)). The mice then received IP injections of the assigned treatment three times a week for a total of 4 weeks. The mice were then sacrificed, and the tumor volume and tumor weight of each tumor were measured. The sizes of the tumors were also measured at the same time. The mice were then sacrificed and the tumors were harvested. All the animal studies were performed under the supervision, guidelines, and approval of the China Medical University Animal Care and Use Committee (#CMOIACUC‐2019‐028).

### 2.11 Statistical analysis

Statistical analyses were performed using the Student's *t*‐test. All of the experiments were repeated at least three times, and a *p* value <0.05 was considered to be statistically significant.

## 3 RESULT

### 3.1 UA is an aromatase silencer in GCa cells

**FIGURE 1:**
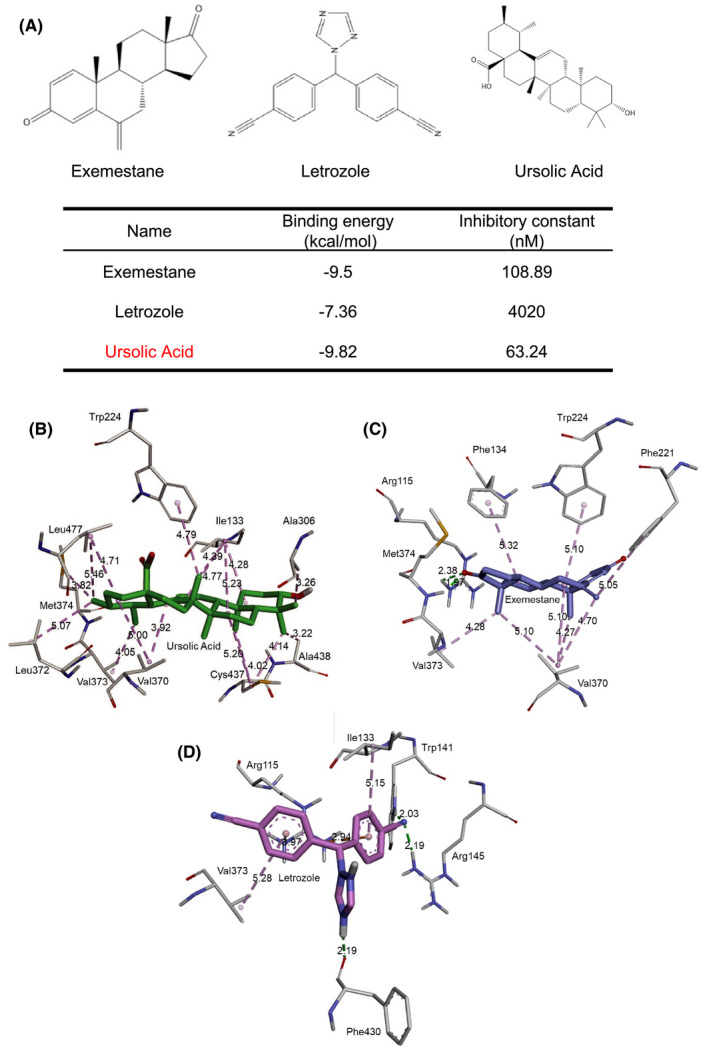
The structures of small molecular exemestane, letrozole, and ursolic acid in binding simulations with CYP19A1/Ar. (A) Structures of exemestane (left‐hand), letrozole (middle), and ursolic acid (right‐hand). The binding energies and Ki values of the compounds are listed in the table below. (B) In one simulation, ursolic acid (green stick) was docked into the active site of human placental aromatase (CYP19A1, PDB ID: 3S7S). Ursolic acid forms a hydrophobic interaction (dashed purple line) with the amino acids (white sticks) Ile133, Trp224, Ala306, Val370, Leu372, Val373, Met374, Cys437, Ala438, and Leu477. (C) In another simulation, exemestane (purple stick) was docked into the active site of human placental aromatase. Exemestane forms hydrogen bonds (dashed green line) with the amino acids Arg115 and Met374. On the other hand, exemestane forms hydrophobic interactions with the amino acids Phe134, Phe221, Trp224, Val370, and Val373. (D) In another simulation, letrozole (pink stick) was docked into the active site of human placental aromatase. Letrozole forms hydrogen bond interactions with the amino acids Trp141, Arg145, and Phe430. Furthermore, it forms hydrophobic interactions with the amino acids Ile133 and Val373. Lastly, it also forms pi–cation charge interactions (dashed orange line) with the amino acid Arg115

In this study, virtual screening with molecular docking calculations was performed to predict the binding affinities between various compounds and aromatase (CYP19A1) in order to identify compounds from the Taiwan Database of Extracts and Compounds (TDEC) that could potentially bind to the active site of aromatase. We ranked the top 10 compounds and compared them with known Ar inhibitors, for example, exemestane and letrozole, and found that UA was among the best in terms of performance in the fitting simulation. It showed that UA exhibited a better binding energy (–9.82 kcal/mol) than exemestane (–9.5 kcal/mol) and letrozole (–7.36 kcal/mol) (Figure 1A).

The docking model showed that UA can successfully dock into the active site of aromatase, and that it then formed 16 hydrophobic alkyl–alkyl interactions with 9 amino acids, namely, Ile133 (4.28 Å, 4.39 Å, 4.77 Å, and 5.23 Å), Ala306 (3.26 Å), Val370 (3.92 Å and 5.00 Å), Leu372 (5.07 Å), Val373 (4.05 Å), Met374 (3.82 Å), Cys437 (4.14 Å, 4.02 Å, and 5.20 Å), Ala438 (3.22 Å), and Leu477 (4.71 Å and 5.46 Å). In addition, it also formed one hydrophobic pi–alkyl interaction with the amino acid Trp224 (4.79 Å). (Figure 1B).

Exemestane can also successfully dock into the active site of aromatase. It formed five hydrophobic alkyl–alkyl interactions with two amino acids, namely, Val370 (4.27 Å, 4.70 Å, 5.10 Å, and 5.10 Å) and Val373 (4.28 Å). In addition, it also formed three hydrophobic pi–alkyl interactions with three amino acids, Phe134 (5.32 Å), Phe221 (5.05 Å), and Trp224 (5.10 Å). Furthermore, it formed two electrostatic hydrogen bond interactions with two amino acids, Arg115 (2.38 Å) and Met374 (1.97 Å). Likewise, its major structure was docked into the location of the co‐crystal exemestane binding space in the active site of aromatase (Figure 1C). On the other hand, the letrozole exhibited a poor docking model (Figure 1D) compared to UA and exemestane. All the interactive distances of all kinds of interactions in every docked compound are shown in parentheses.

**FIGURE 2:**
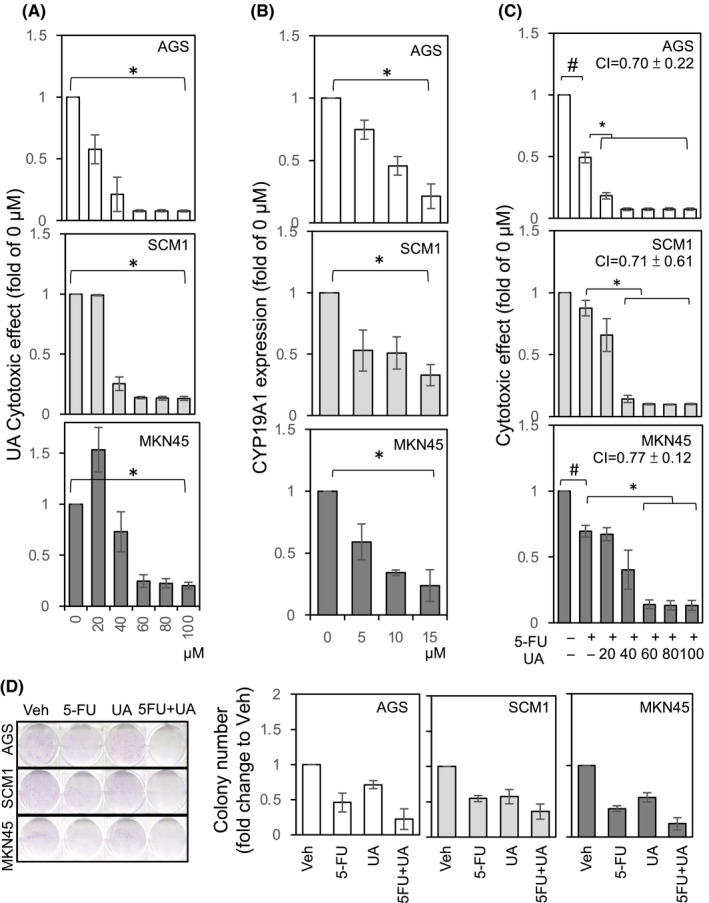
UA suppresses cell growth, silences Ar, and facilitates 5‐FU cytotoxic efficacy in GCa cells. (A) The cytotoxic effect of ARIs was determined using WST‐1 cytotoxicity assays conducted with GCa cells (specifically AGS, SCM‐1, and MKN45 cells). The mean absorbance (450 nm) showed the viability of GCa cells treated with increasing concentrations (0, 20, 40, 60, 80, and 100 μM) of UA for 48 h. (B) Downregulation of Ar by treating GCa cells with UA. GCa cells were treated with UA (0, 5, 10, and 15 μM) for 48 h, and then Ar mRNA was analyzed using qRT‐PCR. (C, D) UA and 5‐FU combined effect was determined with WST‐1 (C) and colony formation (D) assays, and the results showed that the UA and 5‐FU exhibited different efficacy levels in suppressing GCa cell growth with 5‐FU. The CI (combination index) score of UA and 5‐FU combination treatments is labeled on the bar graph. The CI score of three cell lines is: AGS = 0.70 ± 0.22; SCM1 = 0.71 ± 0.61; AGS = 0.77 ± 0.12. * indicates significant differences with *p* values less than 0.05

**FIGURE 3:**
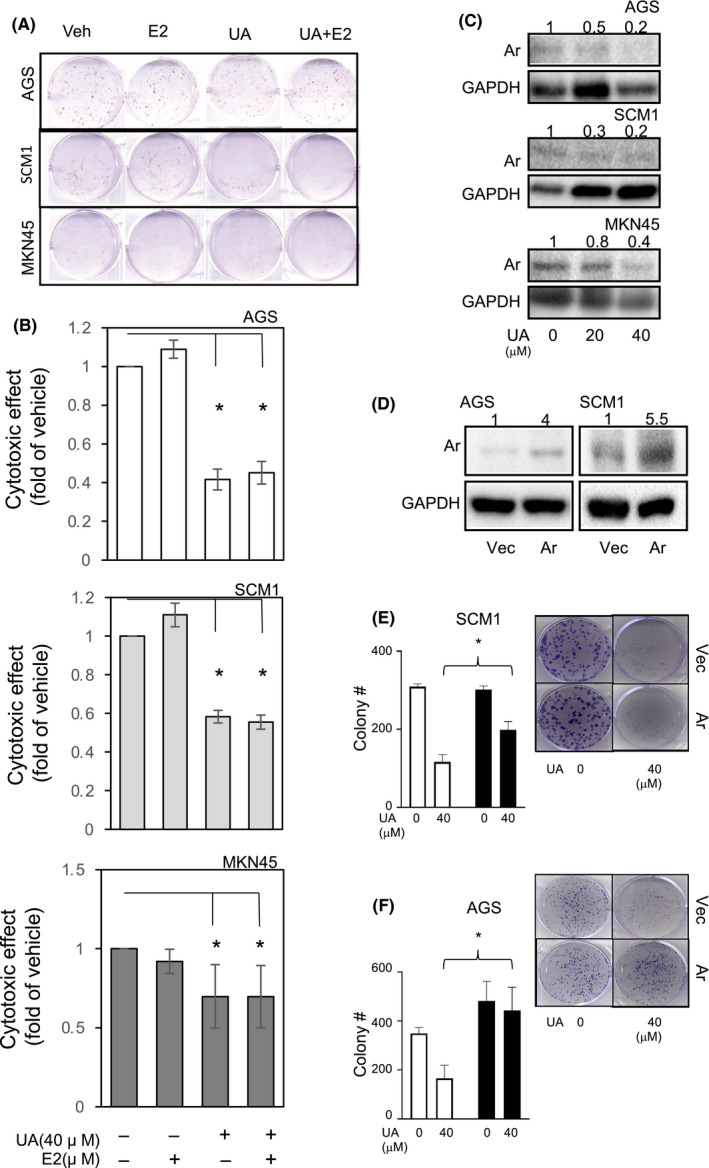
The expression, but not catalytic activity, of Ar affects GCa cell growth. (A) UA or estradiol (E2) was co‐treated on GCa cells to observe their colony‐forming activity. (B) Quantitation of colony of A. * indicates significant differences with *p* values less than 0.05, compared to vehicle treatment. (C) Ar protein expressions upon UA treatments. The UA treatment (‐~40 µM; 24 h) on AGS, SCM1, and MKN45 cells to observe Ar protein expression with immunoblotting assay. The abundance of Ar was balanced with loading control GAPDH with quantitation fold change. (D) Transient expression of Ar cDNA in AGS and SCM1 cells and detected by immunoblot assay. The abundance of Ar was balanced with loading control GAPDH with quantitation fold change. (E, F) Ar reverses UA‐suppressing effect on colony‐forming ability in GCa cells. The Ar cDNA transfection could slightly increase AGS cell colony formatting numbers. As UA 40 µM treatment could robustly suppress colony numbers, Ar cDNA transfection could reverse UA effect on SCM1 (E) and AGS (F) cells. The left‐hand side panels are quantitation of three independent experiments. The right‐hand side panels are representative picture of colony on plate. * denoted significant difference while *p* value less than 0.05

In order to test whether UA exerts a GCa suppression effect, in vitro tests were performed. As shown in Figure 2A, UA treatments exhibited dose‐dependent growth suppression activity on AGS, SC‐M1, and MKN45 GCa cells (with IC.50 < 40 µM). When measuring Ar mRNA expressions, we found that UA can silence Ar expression on GCa cells within 15 µM (Figure 2B). When we co‐treated GCa cells with a low dose of 5‐FU (10 μM)^20^, ^33^ and UA, we found that a synergistic cytotoxic activity occurred on the GCa cells (Figure 2C). To determine whether the drug combination has a synergistic effect or an antagonistic effect. The combination index (CI) of 5‐FU and UA was calculated using CompuSyn software and at Fa 0.5 (50% cell death). Therefore, the combined effect using cytotoxic data generated from the WST‐1 assays. The 5‐FU+UA cotreatment on AGS, SCM1, and MKN45 cells, the CI value is between 0.70 and 0.77, indicating significant synergy of combination effect on the three cell lines. Moreover, as we replicated a similar design in measuring colony‐forming activity, a consistent result was observed (Figure 2D). As shown in Figure 1, UA suppressed cell growth and silenced Ar expression in GCa cells. Considering that the conventional function of Ar is estradiol (E2) production, a physiological relevance E2 dosing (10 nM) was co‐treated on GCa cells to observe the resulting cytotoxic activity of UA. As shown in Figure 3A, adding E2 did not reverse the colony suppression effect of UA with effective cytotoxic dosing (referencing to data in Figure 2A). In addition, E2 co‐treatment of GCa cells with UA to observe their colony‐forming activity showed similar results (Figure 3B). While treated UA on GCa cells, the Ar protein abundance was significantly downregulated (Figure 3C). In order to test if UA colony‐suppressing effect through Ar silencing, the Ar cDNA was transfected in GCa cells (Figure 3D). It appears that restoration of Ar expression in AGS and SCM‐1 cells could significantly reverse UA‐mediated colony‐suppressing effect (Figure 3E,F). However, due to failure to overexpress Ar in MKN45 cells, we exclude this cell line in this experiment.

The data shown in Figures 1, 2, 3 demonstrated the specificity of UA binding on Ar, which resulted in Ar silencing and GCa cell growth suppression.

### 3.2 Potential use of UA, a novel aromatase silencer, for GCA patients

**FIGURE 4:**
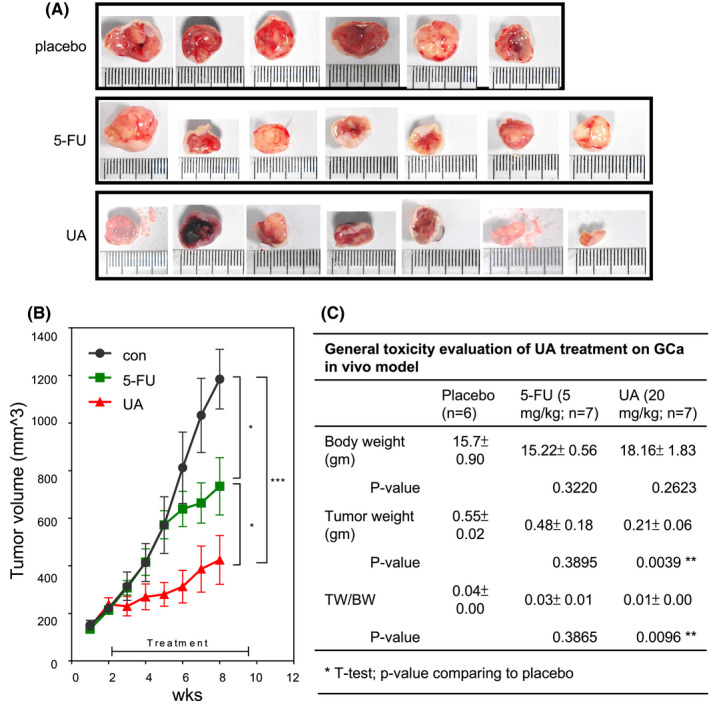
UA or 5‐FU suppresses GCa tumor growth in vivo. (A, B) An MKN45 xenograft mouse model was used to test the tumor‐suppressing effects of UA. A placebo (PBS), 5‐FU (5 mg/kg; low dose), or UA (red line) was intraperitoneally injected three times per week for four consecutive weeks after the tumor size reached 200 mm^3^. The red arrow indicates the time of the initial drug injection. Images of the tumors were obtained when the mice were sacrificed. The data regarding the placebo and 5‐FU effects were obtained from a previously published work,^20^ but conducted simultaneously of different experimental groups. The results for the placebo (black line), low‐dose 5‐FU (green line), and UA (red line) are presented. Astra signs are the *p* values when comparing groups with placebo using ANOVA. (C) Body weight, tumor weight, and tumor weight to body weight ratio of GCa mice at the time of sacrifice. Astra signs are the *p* values when comparing groups with placebo using *t*‐test. *, **, and *** indicate significant differences for *p* values less than 0.05, 0.01, and 0.001, respectively

Moreover, we compared the tumor suppression efficacy of 5‐FU and UA (Figure 4A,B) and evaluated their systemic toxicity (Figure 4C) in MKN45 cells xenografted to create a GCa in vivo model. The 5‐FU treatment using a therapy relevant dosing 5 mg/kg,^31^ where UA was treated in an toxicological acceptable dose 20 mg/kg.^32^ We found that UA exerted extraordinary efficacy (TSI = ~90%) compared to 5‐FU (TSI = ~40%). The tumor weight and tumor weight/body weight (TW/BW) ratio were significantly reduced in the UA‐treated mice compared to the placebo‐treated mice and 5‐FU‐treated mice (Figure 4C). The body weight was comparable in the UA‐treated mice compared to the placebo‐treated mice (*p* = 0.2623).

**FIGURE 5:**
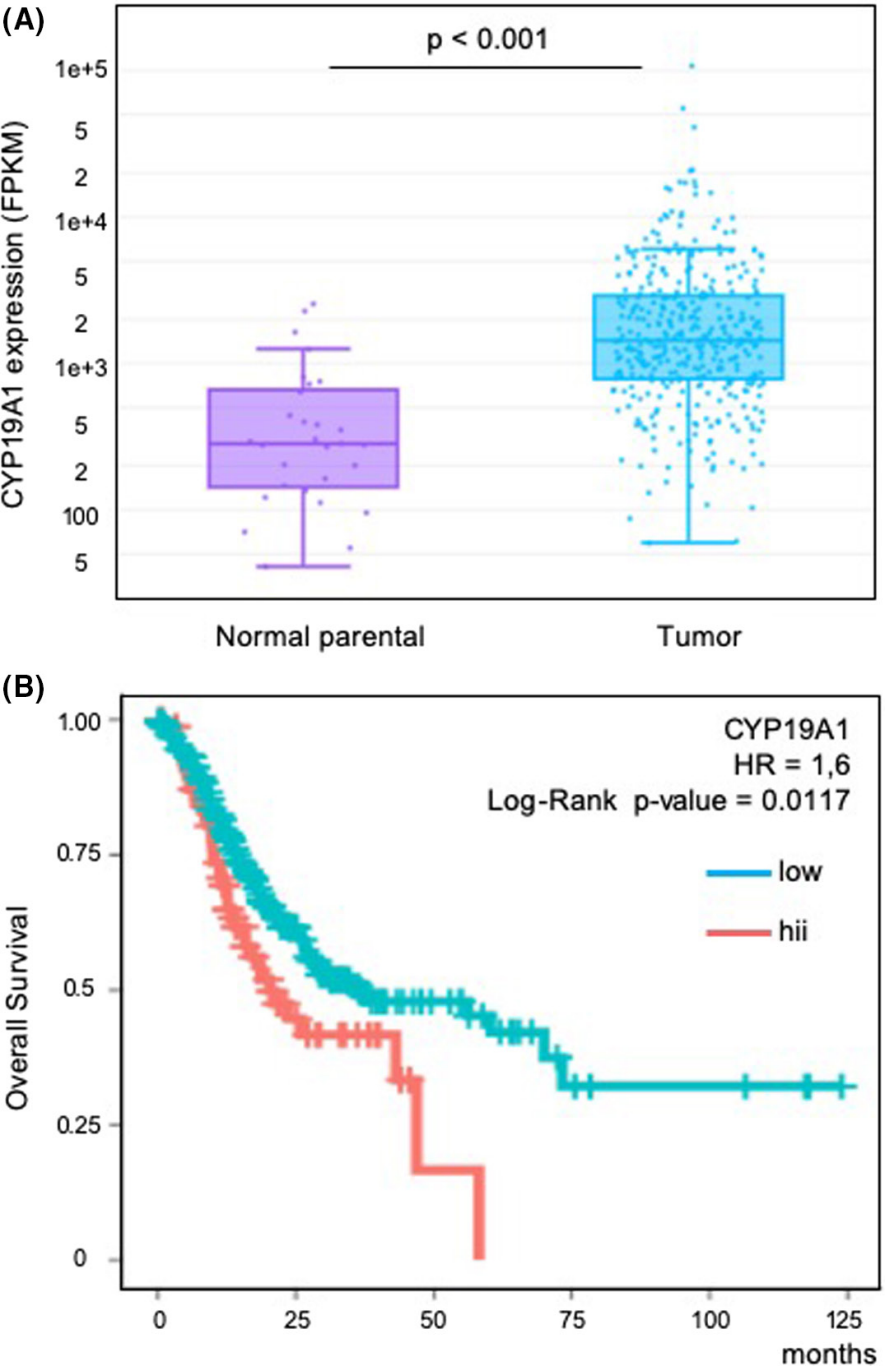
CYP19A1/Ar is a prognostic gatekeeper for GCa patients. (A) The expression comparison of CYP19A1/Ar in normal parental versus GCa tumor lesion of TCGA database cohort. (B) Survival analysis (overall survival) of CYP19A1/Ar mRNA expressions. The red line represents hi (high) expression GCa patients, while the blue line represents lo (low) expression GCa patients. HR, hazard ratio. A log‐rank Kaplan–Meier analysis was performed

In order to survey Ar expression in GCa patients, the TCGA (The Cancer Genome Atlas; https://www.cancer.gov/tcga) database was introduced to observe Ar expressions in a clinical setting. As shown in Figure 5A, the normal parental expression of Ar was lower than that of GCa tumor lesions. This result indicates potential targeting specificity by introducing UA to treat GCa patients. Furthermore, the expression of Ar in GCa tumor lesions is also a prognostic indicator (Figure 5B). These data indicate the potential effectiveness of using UA to silence Ar expression in GCa patients.

**FIGURE 6:**
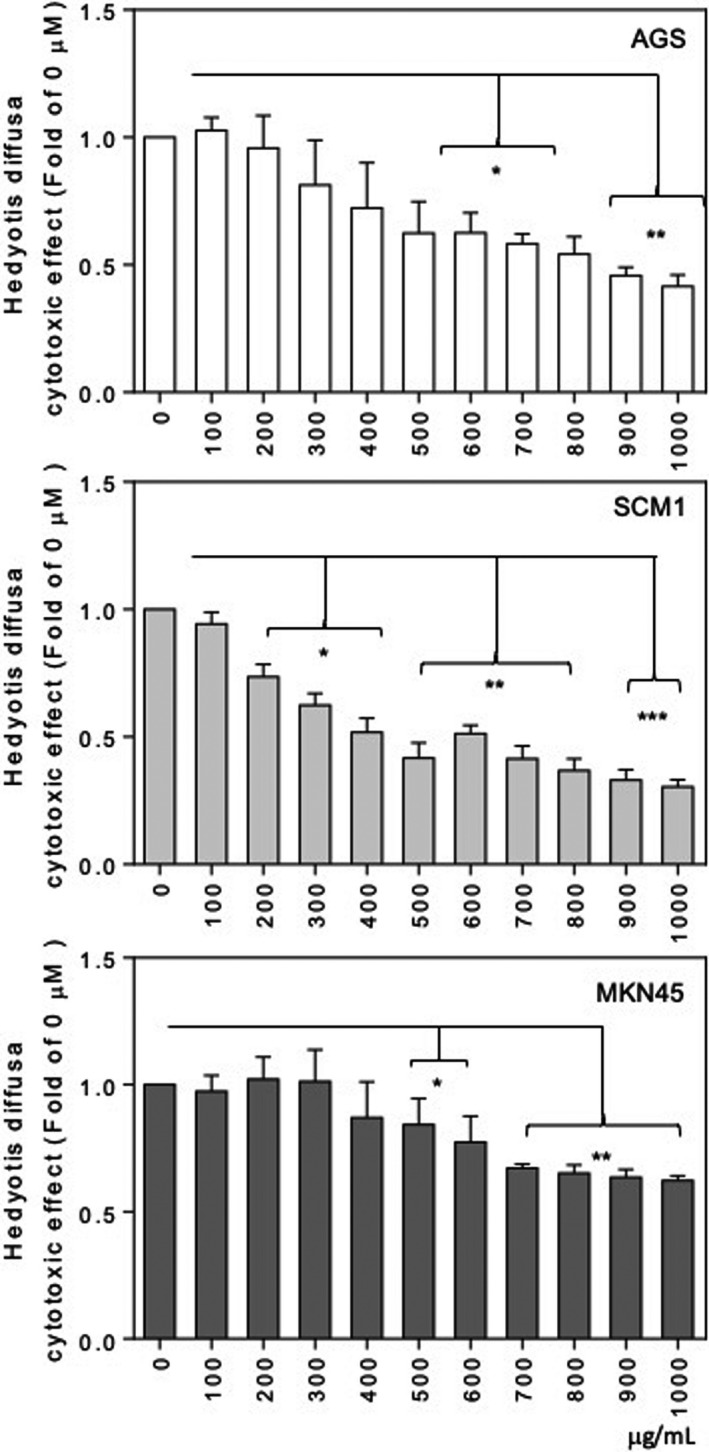
Anti‐GCa activity of *Hedyotis diffusa Willd* ethanol extracts. The cytotoxic effect of ARIs was determined using WST‐1 cytotoxicity assays conducted on GCa cells (AGS, upper; SCM‐1, middle; and MKN45, lower). The mean absorbance (450 nm) showed the viability of GCa cells treated with increasing concentrations (0–1000 μg/ml) of extracts for 48 h

Because UA exerts an anti‐GCa activity via Ar silencing, it could potentially increase the value of UA‐containing natural products used as food supplements, such as *Hedyotis diffusa Willd*, a widely accepted ethnopharmacological natural product for cancer prevention in which UA has been identified as an effective ingredient.^34^, ^35^, ^36^ In this study, therefore, we tested whether *Hedyotis diffusa Willd* ethanol extract could inhibit the viability of GCa cells. In our unpublished data, the batch of crude extract from *Hedyotis diffusa Willd* produced for this report was ~4%. With the administration of *Hedyotis diffusa Willd* ethanol extract onto GCa cells, the cytotoxic efficacy of the extract was shown to be dose‐dependent (Figure 6).

To sum up, the Ar silencing activity of UA might be applied for GCa therapeutics. In addition, *Hedyotis diffusa Willd* ethanol extract was demonstrated to have potential value for the development as a health‐promoting functional food supplement for GCa patients.

## 4 DISCUSSION

**FIGURE 7:**
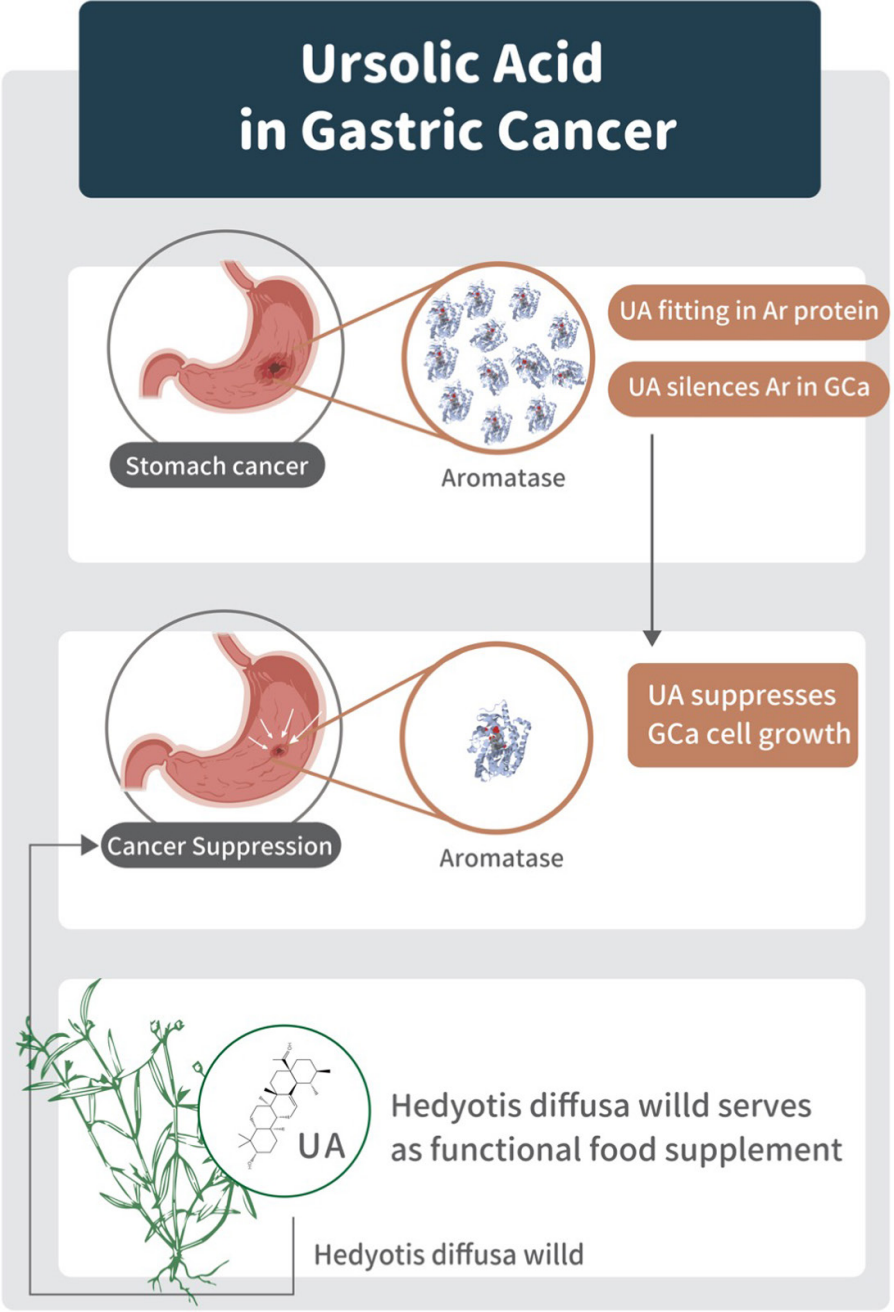
Illustration of UA (also *Hedyotis diffusa Willd* ethanol extracts) suppressing GCa cell growth through the silencing of Ar expression

The results of the current study revealed that UA targets Ar in GCa cells. UA can suppress GCa cells by silencing their Ar expression, which is a prognostic gatekeeper of GCa. In addition, *Hedyotis diffusa Willd* could potentially be introduced as a health‐promoting food supplement. An illustration summarizing the results of this study is presented in Figure 7. The results raise two major issues that should be discussed and are deserving of further study.

### 4.1 AR silencing activity of UA and potential mechanisms

In this study, we found that UA, similar to exemestane, could suppress the cell growth of GCa cells and silence their Ar mRNA expression (Figure 2). A previous study demonstrated that silencing Ar with shRNA^20^ yielded results consistent with those of UA found in this study. That study performed both by Yang et al.^20^ and this work clearly demonstrated a possibility that silencing Ar, in addition of suppressing Ar activity,^37^ could suppress GCa growth. Furthermore, the molecular docking simulations performed in this study revealed that both exemestane and UA exhibited similar MOA in binding with Ar protein (Figure 1). Those data strongly suggested that binding with Ar, presumably on the Trp224 and Met374 of Ar, might turn off the nonenzymatic function of Ar, which could in turn suppress GCa cell growth. Theoretically, there are two potential mechanisms involved in the silencing of Ar activity by UA and exemestane. One would occur through off‐target transcriptional activity suppression, which might not be directly linked to Ar binding. The other would occur through an unidentified negative feedback signaling through which UA or exemestane suppresses the transcriptional activation of Ar.

Regarding the first hypothesis, there have been several studies that support this possibility. For instance, a previous report claimed that GW4064 (farnesoid X receptor agonist) decreased Ar expression in endometriotic cells through ERRK1/2 activation.^38^ Another study showed that megestrol acetate inhibited Ar mRNA expression through nuclear C/EBPβ in an ischemic reperfusion injury rat model.^39^ Such transcriptional suppression on the Ar genome provides good examples indicating that small compounds silence Ar expression transcriptionally.

As for the second hypothesis, there have also been reported examples of such regulation. For example, one study showed that a COX selective inhibitor (sulfonanilide) was surprisingly found to generate selective aromatase modulation activity, in which an aromatic ring was formed with two hydrogen bond acceptors and there was a hydrophobic function of Ar which suppressed Ar mRNA expression.^40^ In another study, methylseleninic acid was found to act as a suppressor of aromatase expression through the direct suppression of the Ar promoter of PI.4‐ and PII‐specific aromatase mRNA expression.^41^ Those are two examples of small compounds suppressing, whether directly on Ar or through an Ar promoter, Ar transcriptional activity.

### 4.2 Potential of UA/*Hedyotis diffusa Willd* as functional food supplement

In this study, we demonstrated that CYP19A1 might be a target for natural products containing ursolic acid, a derivative natural compound from *Hedyotis diffusa Willd*. It was previously reported that *Hedyotis diffusa Willd*, a well‐known Chinese herbal medicine also known as Bai‐Hua‐She‐She‐Ca and as *Oldenlandia diffusa* (Willd), can be used to treat chronic inflammation caused by conditions such as metabolic syndrome, hepatitis, cancer‐related disease.^42^ Notably, *Hedyotis diffusa Willd* has also been used for patients with gastric cancer, and the overall survival analysis showed that *Hedyotis diffusa Willd* improves the outcome of patients with gastric cancer.^43^ According to National Health Insurance Research Database statistics, *Hedyotis diffusa Willd* is the most commonly prescribed Chinese herbal medicine for patients with gastric cancer.^43^ Moreover, phytochemistry studies have revealed that various natural products from *Hedyotis diffusa Willd*, such as triterpenes, flavonoids, and anthraquinones, have been reported to have anticancer activities.^42^ In the current report, the ethanol extracts from *Hedyotis diffusa Willd* were found to be able to suppress GCa cell growth in a dose‐dependent manner. As was noted in the results section, 4% UA was detected in the ethanol extracts of *Hedyotis diffusa Willd*. With regard to industrial application, UA can be easily concentrated for the manufacturing health‐promoting products.

In conclusion, the current study revealed that UA, the anticancer agent from *Hedyotis diffusa Willd* with no identified target, might bind to Ar and, consequently, silence Ar gene transcription and ablate GCa cell growth.

## AUTHOR CONTRIBUTIONS

Wen‐Lung Ma and Ning Chang executed most of the experiments, and drafted the manuscript. Guan‐Yu Chen and Wei‐Chung Cheng are responsible for computational biology. Yang‐Chang Wu is responsible for acquiring the nature product. Ching‐Chia Li and Wei‐Chun Chang advised the translational study and edited the manuscript. Wei‐Chun Chang and Juan‐Cheng Yang supervised the entire project, supported this study, and final approval of the manuscript.

## CONFLICT OF INTEREST

None.

## ETHICAL STATEMENT

This study was not human sample related, therefore, approval from an institutional review board or ethics committee can be waived.

## Data Availability

All the data can bee provided upon reasonable requests to corresponding author.
